# Gestational Age-Dependent Increase of Survival Motor Neuron Protein in Umbilical Cord-Derived Mesenchymal Stem Cells

**DOI:** 10.3389/fped.2017.00194

**Published:** 2017-09-05

**Authors:** Sota Iwatani, Nur Imma Fatimah Harahap, Dian Kesumapramudya Nurputra, Shinya Tairaku, Akemi Shono, Daisuke Kurokawa, Keiji Yamana, Khin Kyae Mon Thwin, Makiko Yoshida, Masami Mizobuchi, Tsubasa Koda, Kazumichi Fujioka, Mariko Taniguchi-Ikeda, Hideto Yamada, Ichiro Morioka, Kazumoto Iijima, Hisahide Nishio, Noriyuki Nishimura

**Affiliations:** ^1^Department of Pediatrics, Kobe University Graduate School of Medicine, Kobe, Japan; ^2^Department of Epidemiology, Kobe University Graduate School of Medicine, Kobe, Japan; ^3^Department of Obstetrics and Gynecology, Kobe University Graduate School of Medicine, Kobe, Japan; ^4^Department of Pathology, Kobe Children’s Hospital, Kobe, Japan; ^5^Department of Developmental Pediatrics, Shizuoka Children’s Hospital, Shizuoka, Japan; ^6^Department of Pediatrics, Hyogo College of Medicine, Nishinomiya, Japan

**Keywords:** spinal muscular atrophy, survival motor neuron-targeted therapy, umbilical cord-derived mesenchymal stem cell, gestational age, perinatal development

## Abstract

**Background:**

Spinal muscular atrophy (SMA) is the most common genetic neurological disease leading to infant death. It is caused by loss of survival motor neuron (SMN) 1 gene and subsequent reduction of SMN protein in motor neurons. Because SMN is ubiquitously expressed and functionally linked to general RNA metabolism pathway, fibroblasts (FBs) are most widely used for the assessment of SMN expression in SMA patients but usually isolated from skin biopsy samples after the onset of overt symptoms. Although recent translational studies of SMN-targeted therapies have revealed the very limited time window for effective SMA therapies during perinatal period, the exact time point when SMN shortage became evident is unknown in human samples. In this study, we analyzed SMN mRNA and protein expression during perinatal period by using umbilical cord-derived mesenchymal stem cells (UC-MSCs) obtained from preterm and term infants.

**Methods:**

UC-MSCs were isolated from 16 control infants delivered at 22–40 weeks of gestation and SMA fetus aborted at 19 weeks of gestation (UC-MSC-Control and UC-MSC-SMA). FBs were isolated from control volunteer and SMA patient (FB-Control and FB-SMA). SMN mRNA and protein expression in UC-MSCs and FBs was determined by RT-qPCR and Western blot.

**Results:**

UC-MSC-Control and UC-MSC-SMA expressed the comparable level of MSC markers on their cell surface and were able to differentiate into adipocytes, osteocytes, and chondrocytes. At steady state, SMN mRNA and protein expression was decreased in UC-MSC-SMA compared to UC-MSC-Control, as observed in FB-SMA and FB-Control. In response to histone deacetylase inhibitor valproic acid, SMN mRNA and protein expression in UC-MSC-SMA and FB-SMA was increased. During perinatal development from 22 to 40 weeks of gestation, SMN mRNA and protein expression in UC-MSC-Control was positively correlated with gestational age.

**Conclusion:**

UC-MSCs isolated from 17 fetus/infant of 19–40 weeks of gestation are expressed functional SMN mRNA and protein. SMN mRNA and protein expression in UC-MSCs is increased with gestational age during perinatal development.

## Introduction

Spinal muscular atrophy (SMA) is a devastating neuromuscular disorder that leads to progressive muscle weakness and atrophy (1, 2). With an estimated disease incidence of 1 in 6,000–10,000 infants, SMA represents the most common lethal genetic disease in infants (3). SMA is caused by the loss of survival motor neuron (SMN) 1 gene (*SMN1*) and retention of highly homologous *SMN2* that differs by only five nucleotides from *SMN1*. As a result of the C-to-T translationally silent transition in *SMN2* exon 7, *SMN2* generates ~90% of exon 7-lacking *SMN* (Δ7-*SMN*) and ~10% of full-length *SMN* (FL-*SMN*) mRNAs, resulting in reduced levels of SMN protein (4, 5).

Patients with SMA are clinically classified into five subtypes based on disease onset and severity: type 0 (the most severe form, prenatal onset), type 1 (severe form; earlier than 6 months old onset, unable to sit unaided), type 2 (intermediate form; earlier than 18 months old onset, unable to stand or walk unaided), type 3 (mild form; later than 18 months old onset, able to stand or walk unaided), and type 4 (the mildest form, adult onset) (6).

An increasing number of translational researches to develop SMN-targeted therapies either targeting endogenous *SMN2* mRNA transcription/splicing or introducing exogenous *SMN1* copies are in progress (2, 7). First clinical trial with histone deacetylase inhibitor valproic acid (VPA) has been shown to increase overall *SMN2* transcription and SMN protein level leading to neuromuscular improvements (8). VPA acts to induce hyperacetylation of histone in the promoter region of *SMN2*, increasing accessibility of the chromatin to transcription factors and machinery and then upregulating *SMN2* transcription. However, subsequent clinical trials with VPA have been completed with only little phenotypic improvements (9, 10).

Antisense oligonucleotides targeting *SMN2*-pre-mRNA (*SMN*-AONs) have achieved long-time rescue of severe SMA mice (*Smn^−/−^SMN2*^+/0^) (11). *SMN*-AONs have been tested in phase III clinical trial in SMA type 1 patients and resulted in the recent approval of nusinersen (Spinraza) as the first drug for SMA patients (12). Intravenous injection of self-complementary serotype 9 adeno-associated virus vectors expressing *SMN1* mRNA on postnatal day 1 successfully rescued neuromuscular function and life span in SMA mice (*Smn^−/−^SMN2*^+/+^*SMN*Δ*7*^+/+^), while injections on postnatal days 5 and 10 resulted in partial correction and little effect, respectively (13). Subsequent SMA mice studies with inducible SMN expression revealed that there was a critical period of time when a sufficient amount of SMN protein was required (14, 15). However, the exact time point when SMN shortage became evident and the actual time window for successful SMN-targeted therapies have remained elusive.

As SMN is ubiquitously expressed and functionally linked to the general RNA metabolism pathway (16–18), cell types other than motor neurons can be used for the assessment of SMN expression in SMA patients. Currently, the most widely used cell type is fibroblasts (FBs) that are usually isolated from skin biopsy samples after the onset of overt symptoms (19).

Mesenchymal stem cells (MSCs) are a heterogeneous population of adherent cells that show potential to proliferate and differentiate into trilineage mesenchymal cells; adipocytes, osteocytes, and chondrocytes. Due to their ability to home to sites of injury, undergo differentiation, suppress immune responses, and modulate angiogenesis, MSCs have significant clinical potential in cell therapies for graft-versus-host disease, myocardial infarction, cerebral infarction, and so on (20–22). Like FBs, MSCs are also easy to isolate and expand from various tissue sources including bone marrow, fat, synovium, periosteum, tooth, cord blood, and umbilical cord (UC). Human UC starts developing with progressive expansion of amniotic cavity at 4–8 weeks of gestation, continue to grow until 50–60 cm in length, and is usually considered medical waste when fetus is delivered. Due to the easy availability without ethical constraints, UC has become a promising source for MSCs (23), Furthermore, UC can be obtained in a non-invasive manner during perinatal period as a result of preterm, term, and postterm delivery.

In the present study, we isolated umbilical cord-derived mesenchymal stem cells (UC-MSCs) from 17 fetus/infants of 19–40 weeks of gestation and analyzed SMN mRNA and protein expression.

## Materials and Methods

### Patients and Samples

Umbilical cords were obtained from 16 control infants (two *SMN1* and two *SMN2* copies, delivered at 22–40 weeks of gestation) and a SMA fetus (no *SMN1* and three *SMN2* copies, aborted at 19 weeks of gestation) with parental written informed consent. Skin biopsy samples were obtained from a control volunteer (two *SMN1* and two *SMN2* copies, skin biopsy at 30 years old) and a type 2/3 SMA patient (no *SMN1* and three *SMN2* copies, skin biopsy at 13 years old) with written informed consent (24). The use of human samples for this study was approved by the ethics committee of Kobe University Graduate School of Medicine (approval no. 1370 and 1694) and conducted in accordance with the approved guidelines.

### Preparation of UC-MSCs

Umbilical cord (about 5 g weight) was collected, washed with PBS, cut into 2- to 3-mm^3^ pieces, enzymatically dissociated with Liberase DH Research Grade (Roche, Mannheim, Germany) in PBS for 45–60 min at 37°C followed by the addition of 10% fetal bovine serum (FBS; Sigma, St. Louis, MO, USA) to inhibit enzyme activity, and filtered through a 100-µm cell strainer (BD Bioscience, Bedford, MA, USA). The resulting cells were cultured at 37°C (5% CO_2_ and 95% air) in MEM-α (Wako Pure Chemical, Osaka, Japan) containing 10% FBS (Sigma) and 1% Antibiotic-antimycotic solution (Invitrogen, Carlsbad, CA, USA) until confluent primary cultures were established. The cells were then disassociated with trypsin-EDTA (Wako Pure Chemical), and the disassociated cells were seeded into fresh dishes and passaged to confluence. Serial passaging was carried out until the tenth passage. The cells at fifth to eighth passage were used in the present experiments.

### Preparation of FBs

Skin biopsy samples were collected, washed with PBS, cut into 1- to 2-mm^3^ pieces, and cultured at 37°C (5% CO_2_ and 95% air) in DMEM (Wako Pure Chemical) containing 10% FBS (Sigma) and 1% Antibiotic-antimycotic solution (Invitrogen). The migrated cells from the minced fragments were grown until sufficient primary cultures were established. The cells were then prepared as described in UC-MSCs.

### Cell Surface Marker Analysis

The cells were dissociated with trypsin-EDTA, washed with PBS, and suspended at ~1 × 10^6^ cells/ml in FCM buffer containing 1× PBS, 2 mM EDTA, and 10% Block Ace (Dainippon Pharmaceutical, Osaka, Japan). The cells were incubated with Fixable Viability Stain 450 (BD Bioscience) for 15 min on ice, washed with PBS, incubated with phycoerythrin-conjugated primary antibodies against CD14, CD19, CD34, CD45, CD73, CD90, CD105, or HLA-DR (all antibodies were purchased from BD Bioscience) for 45 min on ice, washed with PBS, and filtered through a 70-µm cell strainer (BD Bioscience). Flowcytometric analysis was performed using FACSAria III carrying a triple-laser (BD Bioscience).

### Cell Differentiation

For adipogenic differentiation, the cells were cultured in STEMPRO Adipogenesis Differentiation Medium (Invitrogen) for 2–3 weeks and stained with Oil Red O (Sigma). For osteogenic differentiation, the cells were cultured in STEMPRO Osteogenesis Differentiation Medium (Invitrogen) or STK-3 (DS Pharma Biomedical, Osaka, Japan) for 1–2 weeks and stained with Alizarin Red S (Sigma). For chondrogenic differentiation, micromass of the cells were generated, cultured in STEMPRO Chondorogenesis Differentiation Medium (Invitrogen) for 5–7 days, and stained with Toluidine Blue (Sigma). Cell images were acquired using a BZ-X700 microscope (Keyence, Osaka, Japan).

### Quantitative RT-PCR (RT-qPCR)

Total RNA from UC-MSCs and FBs was extracted with a TRIZOL Plus RNA purification kit (Life Technologies, Carlsbad, CA, USA) according to the manufacturer’s instructions. RNA integrity was evaluated by Agilent 2100 Bioanalyzer (Agilent Technologies, Santa Clara, CA, USA) using RNA 6000 Nano kit (Agilent Technologies) according to the manufacturer’s instructions. cDNA was synthesized from 1 µg of total RNA by using a Quantitect Reverse Transcription kit (Qiagen, Valencia, CA, USA). Real-time PCR analysis was performed with an ABI 7500 Real-Time PCR system (Applied Biosystems, Foster City, CA, USA) using FastStart Universal SYBR Green master mix (Roche) with 0.5 µM sense and antisense primers and cDNA (corresponding to 12.5 ng total RNA) according to the manufacturer’s instructions. Each cDNA was amplified with a precycling hold at 95°C for 10 min, followed by 40 cycles at 95°C for 15 s and 60°C for 60 s, and 1 cycle at 95°C for 15 s, 60°C for 60 s, 95°C for 15 s, and 60°C for 15 s. Relative expression of each transcript was calculated based on the ΔΔCt method using glyceraldehyde-3-phosphate dehydrogenase (*GAPDH*) as an endogenous reference for normalization. All sample measurements were repeated at least three times, and the results were expressed as the mean ± SD.

The primer sequences were as follows: for total-*SMN*, the primers were synthesized in the *SMN* exon 1 (5′-AGG AGG ATT CCG TGC TGT TC-3′) and in the *SMN* exon 2b (5′-cgaag GGT TTT TGG TTT ACC CGA AG-3′). For FL-*SMN*, the primers were set in the *SMN* exon 7 (5′-GAA GGT GCT CAC ATT CCT TAA AT-3′) and in the *SMN* exon 8 (5′-ATC AAG AAG AGT TAC CCA TTC CA-3′) (25). For Δ7-*SMN*, the primers were designed in the *SMN* exon 5 (5′- CCA CCA CCC CAC TTA CTA TCA-3′) and at the *SMN* exon6/exon8 border (5′-GCT CTA TGC CAG CAT TTC CAT A-3′) (25). For *GAPDH*, the primers were synthesized at the *GAPDH* exon 2/3 border (5′-GAG TCA ACG GAT TTG GTC GT-3′) and in the *GAPDH* exon 4 (5′-GAC AAG CTT CCC GTT CTC AG-3′) (26).

### Western Blot

Umbilical cord-derived mesenchymal stem cells and FBs were lysed with RIPA buffer [50 mM Tris/HCl (pH 7.5) containing 1.0% NP-40, 1.0% sodium deoxycholate, 0.1% SDS, 150 mM NaCl, and 1 mM EDTA]. Cell lysates were separated by SDS-PAGE, and proteins were transferred to polyvinylidene difluoride membranes (Millipore). Membranes were blocked with Block Ace (Dainippon Pharmaceutical), incubated with mouse anti-SMN (1:1,000; BD Transduction Laboratories, Franklin Lakes, NJ, USA) or mouse anti-beta-actin (Abcam, Cambridge, MA, USA; 1:1,000), incubated with horseradish peroxidase (HRP)-coupled anti mouse IgG (1:100,000; Jackson ImmunoResearch Laboratories, West Grove, PA, USA), and developed by Immobilon Western Chemiluminescent HRP Substrate (Millipore). Chemiluminescent signals were captured by Amersham Imager 600 (GE Healthcare Biosciences, Pittsburgh, PA, USA) according to the manufacturer’s instructions. Relative SMN expression was calculated based on the beta-actin expression.

### Statistical Analysis

Pearson’s correlation coefficients were determined, and the Mann–Whitney *U* test was used to compare two independent data sets, using Excel software (Microsoft, Redmond, WA, USA) and Excel Statistics (Statcel 3; Social Survey Research Information Co., Tokyo, Japan). Differences were considered statistically significant for *P* < 0.05.

## Results

### Isolation of UC-MSC-Control and UC-MSC-SMA

**Figure 1:**
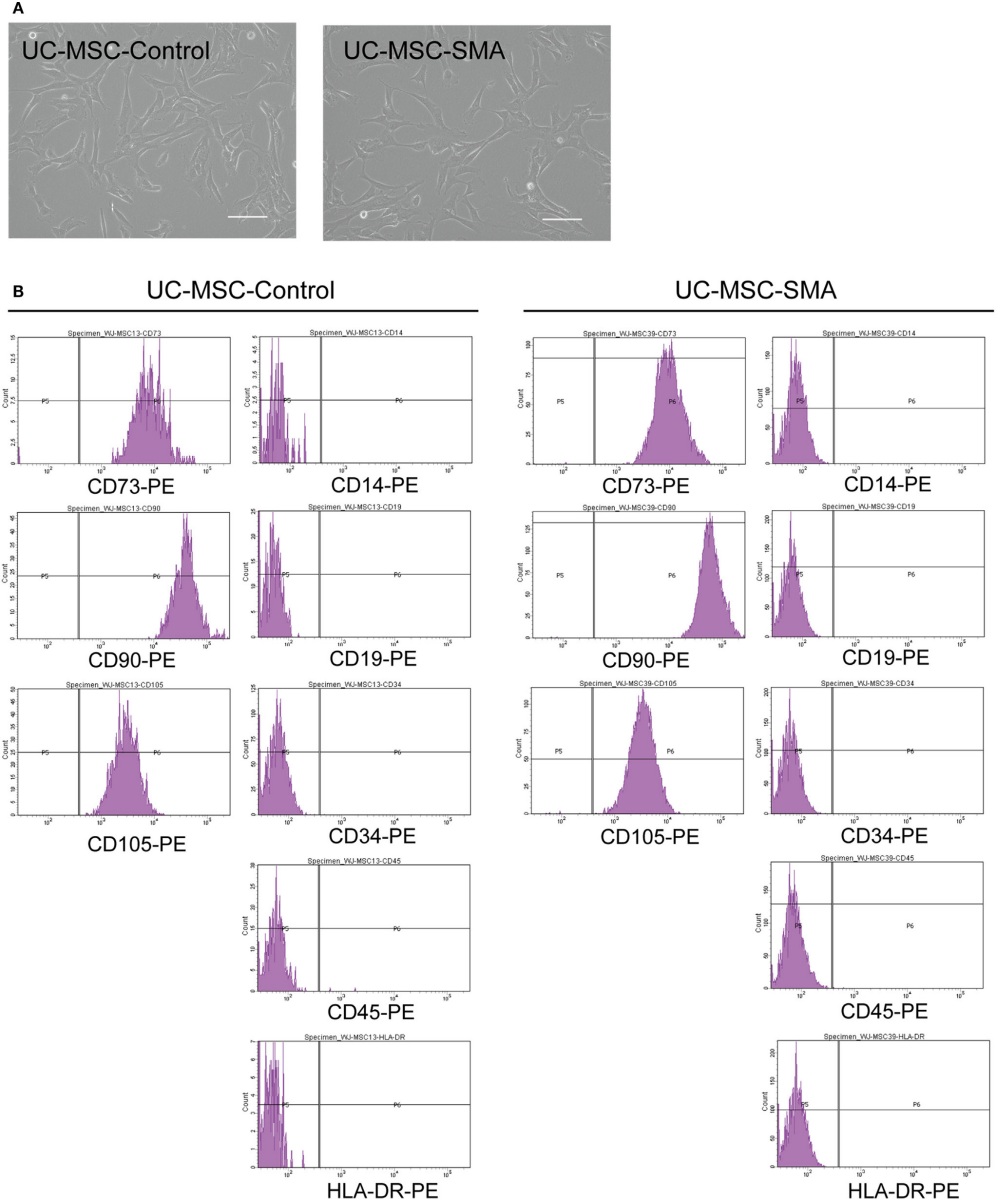
Cell morphology and surface markers expression of umbilical cord-derived mesenchymal stem cell (UC-MSC)-Control and UC-MSC-spinal muscular atrophy (SMA). **(A)** UC-MSCs from control infant (UC-MSC-Control; 22 weeks of gestation) and aborted SMA fetus (UC-MSC-SMA; 19 weeks of gestation) were examined by phase-contrast microscopy. The images shown are representative of three independent experiments. Scale bars show 100 µm. **(B)** UC-MSC-Control and UC-MSC-SMA were analyzed by flowcytometer using antibodies against MSC markers (CD14, CD19, CD34, CD45, CD73, CD90, CD105, and HLA-DR) defined by ISCT (27). The histograms shown are representative of three independent experiments.

**Figure 2:**
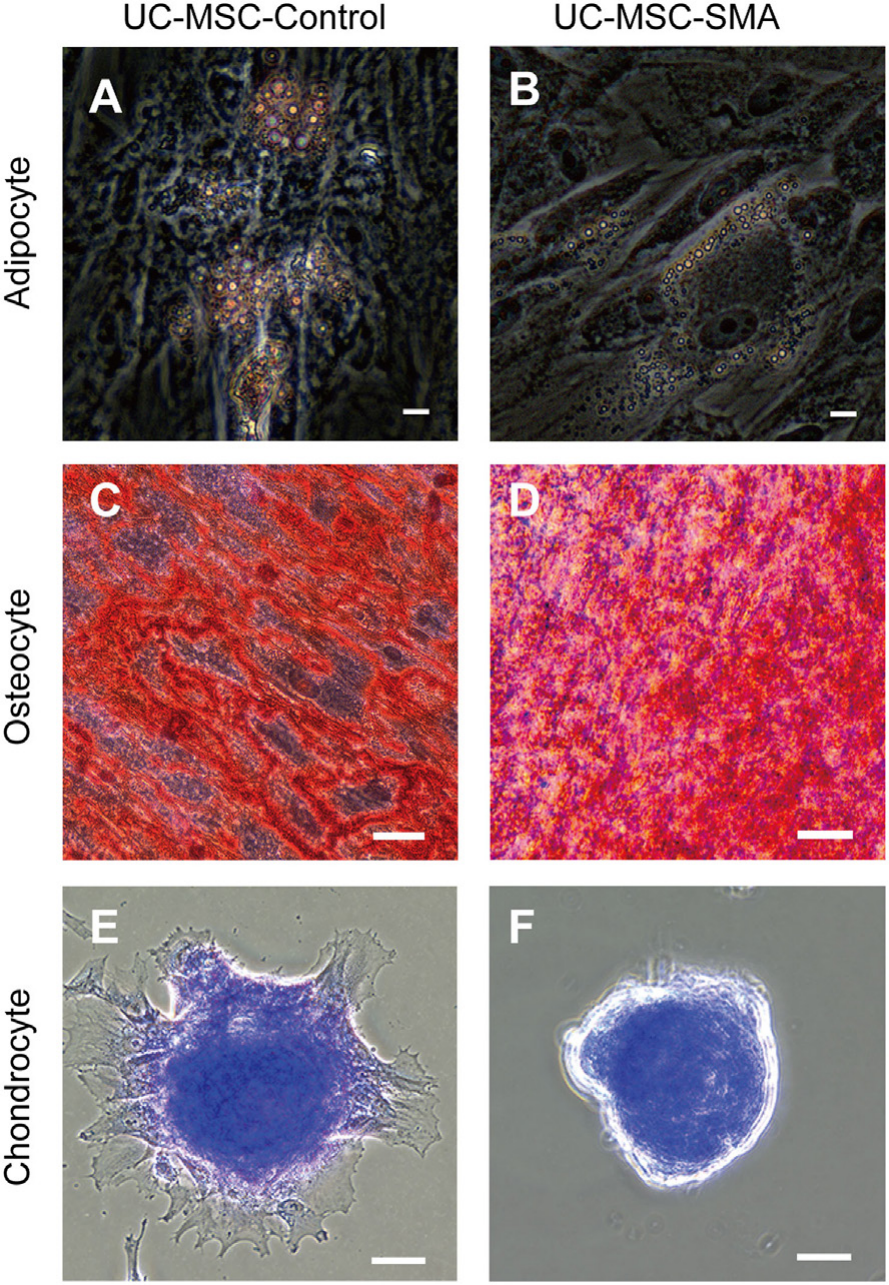
Trilineage mesenchymal differentiation of umbilical cord-derived mesenchymal stem cell (UC-MSC)-Control and UC-MSC-spinal muscular atrophy (SMA). UC-MSC-Control and UC-MSC-SMA were differentiated into adipocyte as visualized by Oil Red O [**(A,B)**, scale bars: 10 µm], into osteocyte as visualized by Alizarin Red S [**(C,D)**, scale bars: 50 µm], and chondrocyte as visualized by Toluidine Blue [**(E,F)**, scale bars: 50 µm]. The images shown are representative of three independent experiments.

To determine the exact time point when SMN shortage became evident in human samples, we first isolated MSCs from UC of control infant delivered at 22 weeks and SMA fetus aborted at 19 weeks of gestation (UC-MSC-Control and UC-MSC-SMA). Both UC-MSC-Control and UC-MSC-SMA exhibited a spindle-like shape (Figure 1A) and were positive for CD73, CD90, and CD105, but negative for CD14, CD19, CD34, CD45, and HLA-DR (Figure 1B). Under standard *in vitro* differentiation conditions, UC-MSC-Control and UC-MSC-SMA were comparably differentiated into osteocytes, adipocytes, and chondrocytes (Figure 2). Collectively, UC-MSC-Control and UC-MSC-SMA fulfill the criteria for MSC defined by ISCT position paper (27).

### SMN mRNA and Protein Expression in UC-MSCs and FBs at Steady State

**Figure 3:**
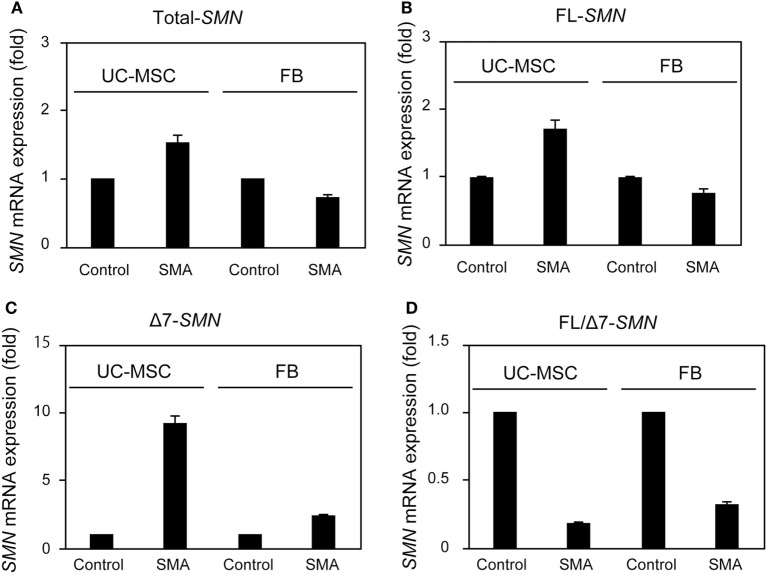
Survival motor neuron (*SMN*) mRNA expression in umbilical cord-derived mesenchymal stem cells (UC-MSCs) and fibroblasts (FBs) at steady state. Relative expression of total-*SMN* **(A)**, FL-*SMN* **(B)**, and Δ7-*SMN* **(C)** mRNA in UC-MSC-Control, UC-MSC-spinal muscular atrophy (SMA), FB-Control, and FB-SMA was analyzed by RT-qPCR and FL/Δ7-*SMN* ratio **(D)** was calculated. The expression in UC-MSC-Control and FB-Control was defined as 1. The results were expressed as the mean ± SD.

To examine SMN expression in UC-MSC-Control and UC-MSC-SMA at steady state, we isolated FBs from control volunteer (FB-Control; 30 years old) and SMA patient (FB-SMA; 13 years old) as a reference. Total- and FL-*SMN* mRNA expression was comparable among FB-Control, FB-SMA, UC-MSC-Control, and UC-MSC-SMA (Figures 3A,B). In contrast, Δ7-*SMN* mRNA expression in FB-SMA and UC-MSC-SMA trended to be higher than FB-Control and UC-MSC-Control, respectively (Figure 3C). Accordingly, FL/Δ7-*SMN* ratio in FB-SMA and UC-MSC-SMA trended to be lower than FB-Control and UC-MSC-Control, respectively (Figure 3D).

**Figure 4:**
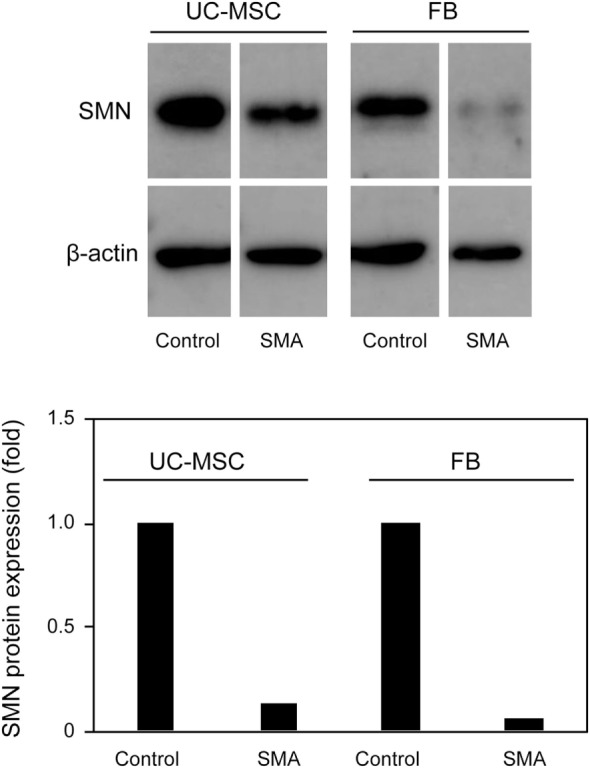
Survival motor neuron (SMN) protein expression in umbilical cord-derived mesenchymal stem cells (UC-MSCs) and fibroblasts (FBs) at steady state. Relative expression of SMN protein in UC-MSC-Control, UC-MSC-spinal muscular atrophy (SMA), FB-Control, and FB-SMA was analyzed by Western blot. The images shown are representative of three independent experiments. The expression in UC-MSC-Control and FB-Control was defined as 1.

Survival motor neuron protein expression was markedly decreased in FB-SMA compared to FB-Control as described previously (Figure 4) (26). Like FB-SMA, UC-MSC-SMA also trended to express the lower level of SMN protein than UC-MSC-Control. Notably, the easily detectable level of SMN protein was expressed in UC-MSC-SMA in contrast to the barely detectable expression in FB-SMA (Figure 4). These results suggest that SMN mRNA and protein expression in UC-MSCs is compatible with FBs at steady state.

### SMN mRNA and Protein Expression in UC-MSCs and FBs in Response to VPA

**Figure 5:**
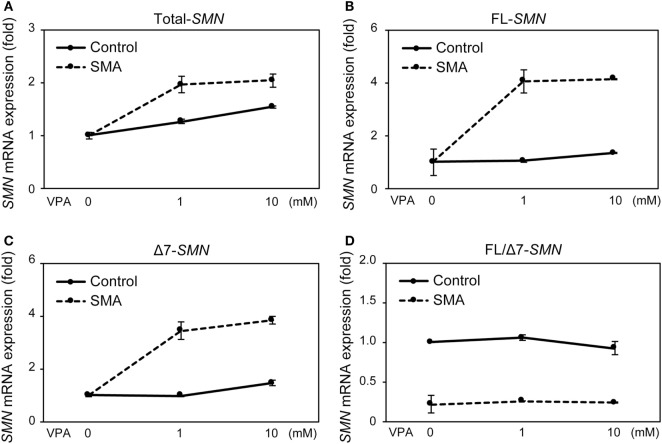
Survival motor neuron (*SMN*) mRNA expression in umbilical cord-derived mesenchymal stem cells (UC-MSCs) and fibroblasts in response to valproic acid (VPA). UC-MSC-Control and UC-MSC-spinal muscular atrophy (SMA) were treated with 0, 1, and 10 mM VPA for 16 h and relative expression of total-*SMN* **(A)**, full-length (FL)-*SMN* **(B)**, and Δ7-*SMN* **(C)** mRNA was analyzed by RT-qPCR and FL/Δ7-*SMN* ratio **(D)** was calculated. The expression in 0 mM VPA was defined as 1.

**Figure 6:**
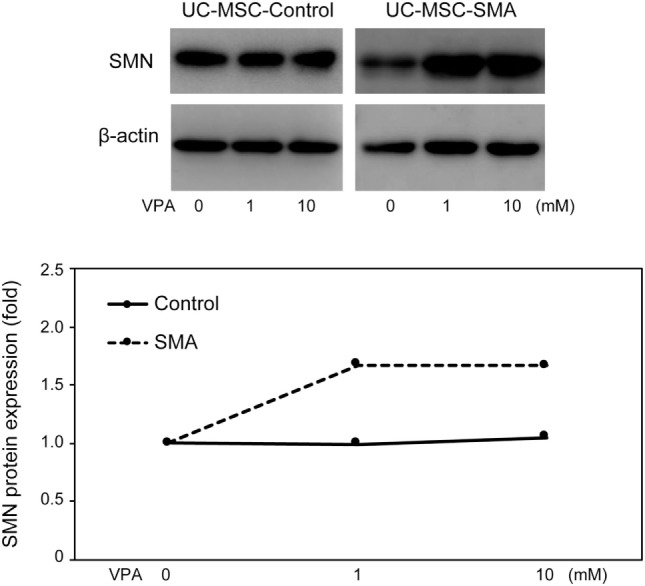
Survival motor neuron (SMN) protein expression in umbilical cord-derived mesenchymal stem cells (UC-MSCs) and fibroblasts in response to valproic acid (VPA). UC-MSC-Control and UC-MSC-spinal muscular atrophy (SMA) were treated with 0, 1, and 10 mM VPA for 16 h and relative expression of SMN protein was analyzed by Western blot. The images shown are representative of three independent experiments. The expression in 0 mM VPA was defined as 1.

Valproic acid (VPA) is known to increase SMN mRNA and protein expression in FB-SMA (26, 28, 29). To examine the functionality of SMN mRNA and protein expression in UC-MSCs, the effect of VPA on SMN expression was compared between UC-MSCs and FBs. UC-MSC-Control and UC-MSC-SMA were treated with 0, 1, and 10 mM VPA for 16 h and total-, FL-, and Δ7-*SMN* mRNA expression was determined by RT-qPCR. Total-, FL-, and Δ7-*SMN* mRNA expression in UC-MSC-Control trended to be increased upon VPA treatment. VPA treatment trended to show more profound effect on the expression of total-, FL-, and Δ7-*SMN* mRNA expression in UC-MSC-SMA compared to UC-MSC-Control (Figures 5A–C). In contrast, FL/Δ7-*SMN* ratio in both UC-MSC-Control and UC-MSC-SMA was almost constant upon VPA treatment (Figure 5D). In terms of SMN protein expression, UC-MSC-SMA trended to upregulate SMN protein expression on VPA treatment, while UC-MSC-Control showed little response to VPA treatment (Figure 6). These results suggest that SMN mRNA and protein expression in UC-MSCs and FBs was comparably increased in response to VPA.

### SMN mRNA and Protein Expression in UC-MSCs from Control Infants Delivered at Various Gestational Age

**Figure 7:**
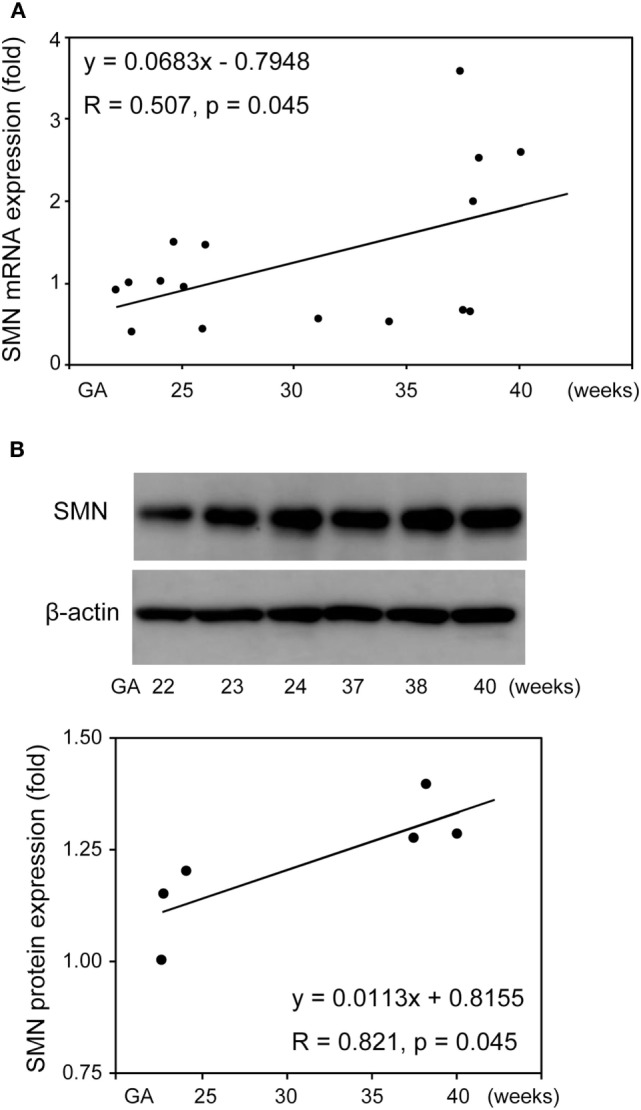
Survival motor neuron (SMN) mRNA and protein expression in umbilical cord-derived mesenchymal stem cells (UC-MSCs) from control infants delivered at various gestational age. Relative expression of full-length-*SMN* mRNA **(A)** and SMN protein **(B)** in UC-MSCs from 16 and 6 control infants (22–40 gestational age) was analyzed by RT-qPCR and Western blot, respectively. The expression in 22 weeks of gestation was defined as 1. The results were expressed as the mean, and the images shown are representative of three independent experiments.

Given that SMN mRNA and protein was functionally expressed in UC-MSCs as well as FBs, we determined SMN mRNA and protein expression in UC-MSCs from various gestational age infants. Total RNA of 16 UC-MSCs from control infants delivered at 22–40 weeks of gestation was extracted and subjected to RT-qPCR. There was significant positive correlation between FL-*SMN* mRNA expression and gestational age in these 16 UC-MSCs (*R* = 0.507, *P* = 0.045; Figure 7A). Protein lysate was then prepared from six UC-MSCs and subjected to Western blot. Positive association between SMN protein expression and gestational age was also detected in UC-MSCs (*R* = 0.821, *P* = 0.045, Figure 7B). Taken together, SMN mRNA and protein expression in UC-MSCs is increased with gestational age.

## Discussion

In this study, we isolated UC-MSCs from 16 control infants delivered at 22–40 weeks of gestation and SMA fetus aborted at 19 weeks of gestation. These UC-MSCs functionally expressed SMN mRNA and protein and compatible with FBs (19, 26, 28, 29). We then demonstrated that SMN mRNA and protein expression in UC-MSCs was increased with gestational age. To our knowledge, this is the first study to reveal the SMN protein expression during perinatal development in human samples.

There is increasing evidence that other cell types than motor neuron contribute SMA phenotypes (16–18). Currently, FBs from skin biopsy samples have been widely used for the assessment of SMN expression in SMA patients, due to their easy accessibility (19). Because FBs are usually isolated after the onset of overt symptoms, we isolated UC-MSCs to overcome this time limitation of FBs and carefully assessed whether UC-MSCs were compatible with FBs for the assessment of SMN expression in SMA patients. Consistent with FBs, UC-MSC-SMA expressed ~1.6 times more FL-*SMN* mRNA and ~5 times less SMN protein than UC-MSC-Control at steady state (Figures 3C and 4). In accordance with FBs, an approximately 3.3-fold increase of FL-*SMN* mRNA and 1.8-fold increase of SMN protein was detected in UC-MSC-SMA compared to UC-MSC-Control in response to 1 mM VPA treatment for 16 h (Figures 5B and 6). Notably, the effective concentration of VPA in UC-MSC-SMA was considerably lower than that in FB-SMA (26). This study demonstrated that UC-MSCs functionally expressed SMN mRNA and protein.

We then determined SMN expression in UC-MSCs obtained at 19–40 weeks of gestation, reflecting a part of human perinatal development. In contrast to the previous observations reporting a marked reduction of SMN protein expression in human brain, skeletal muscle, and kidney after birth (30), the present study revealed a significant increase of SMN expression (~3.0 fold FL-*SMN* mRNA and ~1.2 fold SMN protein) during perinatal period of human development (Figure 7). Collectively, it is tempting to speculate that human SMN expression is increased during perinatal period and decreased afterward. If this is the case, human SMN shortage may become evident after 40 weeks of gestation. Further elucidation of human SMN expression in the perinatal period of control and SMA patients will set the foundation for curative SMA treatment.

In summary, UC-MSCs expressing functional SMN mRNA and protein are successfully isolated from 17 fetus/infants of 19–40 weeks of gestation. SMN mRNA and protein expression in UC-MSCs is increased with gestational age during perinatal development.

## Ethics Statement

The use of human samples for this study was approved by the ethics committee of Kobe University Graduate School of Medicine (approval no. 1370 and 1694), and conducted in accordance with the approved guidelines.

## Author Contributions

SI, HN, and NN drafted the initial manuscript; conceptualized and designed the study. SI, NH, DN, ST, AS, DK, KY, KT, and TK collected the samples, acquired the data, and carried out the analyses. SI and NN performed statistical analysis and interpreted the data. SI, MY, MM, KF, MT-I, HY, IM, KI, HN, and NN revised the article critically for important intellectual content.

## Conflict of Interest Statement

The authors declare that the research was conducted in the absence of any commercial or financial relationships that could be construed as a potential conflict of interest.
